# Body size and substrate type modulate movement by the western Pacific crown-of-thorns starfish, *Acanthaster solaris*

**DOI:** 10.1371/journal.pone.0180805

**Published:** 2017-09-06

**Authors:** Morgan S. Pratchett, Zara-Louise Cowan, Lauren E. Nadler, Ciemon F. Caballes, Andrew S. Hoey, Vanessa Messmer, Cameron S. Fletcher, David A. Westcott, Scott D. Ling

**Affiliations:** 1 ARC Centre of Excellence for Coral Reef Studies, James Cook University, Townsville, Australia; 2 Scripps Institution of Oceanography, University of California San Diego, La Jolla, CA, United States of America; 3 CSIRO Land and Water, Atherton, Queensland, Australia; 4 Institute for Marine and Antarctic Studies, University of Tasmania, Hobart, Australia; Department of Agriculture and Water Resources, AUSTRALIA

## Abstract

The movement capacity of the crown-of-thorns starfishes (*Acanthaster* spp.) is a primary determinant of both their distribution and impact on coral assemblages. We quantified individual movement rates for the Pacific crown-of-thorns starfish (*Acanthaster solaris*) ranging in size from 75–480 mm total diameter, across three different substrates (sand, flat consolidated pavement, and coral rubble) on the northern Great Barrier Reef. The mean (±SE) rate of movement for smaller (<150 mm total diameter) *A*. *solaris* was 23.99 ± 1.02 cm/ min and 33.41 ± 1.49 cm/ min for individuals >350 mm total diameter. Mean (±SE) rates of movement varied with substrate type, being much higher on sand (36.53 ± 1.31 cm/ min) compared to consolidated pavement (28.04 ± 1.15 cm/ min) and slowest across coral rubble (17.25 ± 0.63 cm/ min). If average rates of movement measured here can be sustained, in combination with strong directionality, displacement distances of adult *A*. *solaris* could range from 250–520 m/ day, depending on the prevailing substrate. Sustained movement of *A*. *solaris* is, however, likely to be highly constrained by habitat heterogeneity, energetic constraints, resource availability, and diurnal patterns of activity, thereby limiting their capacity to move between reefs or habitats.

## Introduction

The rate at which animals move has a major bearing on their ecology, influencing their exposure to different environments, foraging patterns, and biological interactions [1,2]. Short-term maximum rates of movement influence prey capture and/or predator avoidance, whereas sustained rates of movement affect the animal’s capacity to move between different environments and/or habitat patches [1]. Ultimately, this locomotor capacity influences the fate of individuals, the structure of populations, and the evolution of species [2]. Studies of animal movement (of both rates and patterns of movement) are important to understand temporal dynamics in the distribution of individuals and populations [3,4], and their corresponding interactions and impacts across environmental gradients and habitats.

Crown-of-thorns starfishes (*Acanthaster* spp.) are notorious for the damage they cause within coral reef environments [5]. Most notably, periodic outbreaks of *Acanthaster* spp. rapidly deplete local coral assemblages [6,7] and are a major contributor to sustained coral loss across the Indo-Pacific [8,9]. During severe outbreaks, crown-of-thorns starfishes may form feeding fronts that systematically remove prey corals as they move [10]. The locomotor capacity of crown-of-thorns starfishes, both in terms of direction and velocity, will, therefore, influence the patterns and spatial extent of their impacts [6,11]. The capacity of these starfishes to move within and among reef habitats is also fundamental in addressing long-standing controversies surrounding the initiation and spread of population outbreaks. Moreover, understanding the spatial and temporal scales at which adult starfishes move in the landscape will inform the spatial and temporal scales required to design effective and efficient control programs [12].

Echinoderms have a unique biomechanical mechanism for locomotion, using their water-vascular system to control the extension of numerous and largely independent podia [13]. For asteroids, the oral side of the animal attaches to the substrate using many tubular podia (hereafter referred to as tube feet), which inflate, retract and extend using a hydrostatic canal system to initiate crawling [14]. The maximum recorded movement rates of adult *Acanthaster* spp. range from 33.3 cm min^–1^ [15], up to 50.9 cm min^–1^ [16], though the latter was recorded over an artificial (plastic) substrate. Among natural reef substrates, *Acanthaster* spp. move fastest over sand, where average maximum velocity (33.3 cm min^–1^; [15]) is almost twice that recorded for other reef substrates [17]. However, crown-of-thorns starfishes placed on sand rapidly move towards nearby physical habitat structures [18,19]. Thus, apparent differences in movement rates on different substrates may reflect differential motivations for movement, rather than the physiological capabilities of *Acanthaster* spp. When moving among continuous reef habitats, crown-of-thorns starfishes typically exhibit semi-diurnal cycles of activity, and spend <40% of time actually moving [20]. Over longer periods (days to weeks), maximum time-averaged rates of movement for adults are only 2.50–5.75 cm/ min [17], suggesting that crown-of-thorns starfishes do not sustain high rates of movement for more than a few hours. Patterns of activity, and therefore rates of sustained movement, may be further constrained by the recent feeding history and physiological condition of individual starfish, though *Acanthaster* spp. spend more time moving (cf. feeding) in the absence of prey [20], and starvation can actually initiate movement [21].

Locomotor capacity of echinoderms is expected to increase with body size, due to corresponding increases in the number and/ or size of tube feet [16], though the actual relationship with body size appears to be highly species-specific [16,22]. For crown-of-thorns starfishes, Mueller et al. [16] suggested that there was no effect of body size on movement rates. However, meta-data compiled by Moran [17] clearly show a distinct difference in the locomotor capacity of juvenile versus adult crown-of-thorns starfishes. Importantly, Yamaguchi [23] showed that newly settled crown-of-thorns starfishes moved very slowly (<0.01 cm/ min), which would greatly constrain their capacity for movement between discrete habitat types. It has been suggested however, that crown-of-thorns starfish settle in deep-water habitats and migrate to shallow-water, coral-dominated habitats, following ontogenetic shifts in feeding behaviour [24].

The purpose of this study was to explicitly test for size- and substrate-specific differences in rates of movement (mean and maximum velocity) for the Pacific species of crown-of-thorns starfish, *Acanthaster solaris* [25]. While several previous studies have quantified movement rates of *Acanthaster* spp. (reviewed by [17]), most estimates are based on hourly or daily displacement under field conditions, with no account of the specific substrate type (e.g. [26]). We conducted a controlled laboratory experiment, explicitly testing for effects of body size and substrate type on locomotor capacity of *A*. *solaris*. We hypothesized that larger individuals would exhibit greater mean and maximum movement rates, especially compared to the smaller (juvenile) starfish. Given that increased substrate complexity effectively increases the distance over which starfish must travel to achieve the same horizontal displacement [27] and increases the probability and proportion of tube feet not in contact with the substrate at any point in time, we also expect that movement would be much more constrained over rubble, compared to relatively flat carbonate pavement or sand. Quantifying movement rates for *Acanthaster* spp. across different reef substrates is important, not only for understanding the spread and progression of their impacts, but also for optimising spatial and temporal aspects of population control (or culling) activities [28], such that these findings will have important management implications.

## Methods

### Ethics statement, sampling and maintenance

This study was carried out in strict accordance with the guidelines set out by James Cook University and the Lizard Island Research Station. Collection of crown-of-thorns starfish was conducted under Great Barrier Reef Marine Park Authority (GBRMPA) Permit No.G15/37363.1. *Acanthaster solaris* were collected at Lizard Island (14^o^40’S, 145^o^27’E) and surrounding reefs (mainly Eyrie Reef) in the northern Great Barrier Reef (GBR), Australia, in November 2015. Coral cover at the specific collection sites was >15%, ensuring that starfish still had reasonable access to prey corals (including *Acropora*). Starfish were collected on snorkel or scuba (depending on working depths), using long (40 cm) purpose-built stainless wire tongs to extract starfish from the reef matrix, while taking care to ensure that starfish were not damaged during collection or transport. Starfish ranged in size from 75 mm to 490 mm total diameter. All starfish were held in captivity in 1000-L plastic aquaria with high throughput of fresh seawater for a maximum of 6 days prior to use in movement trials.

### Morphology

**Fig 1:**
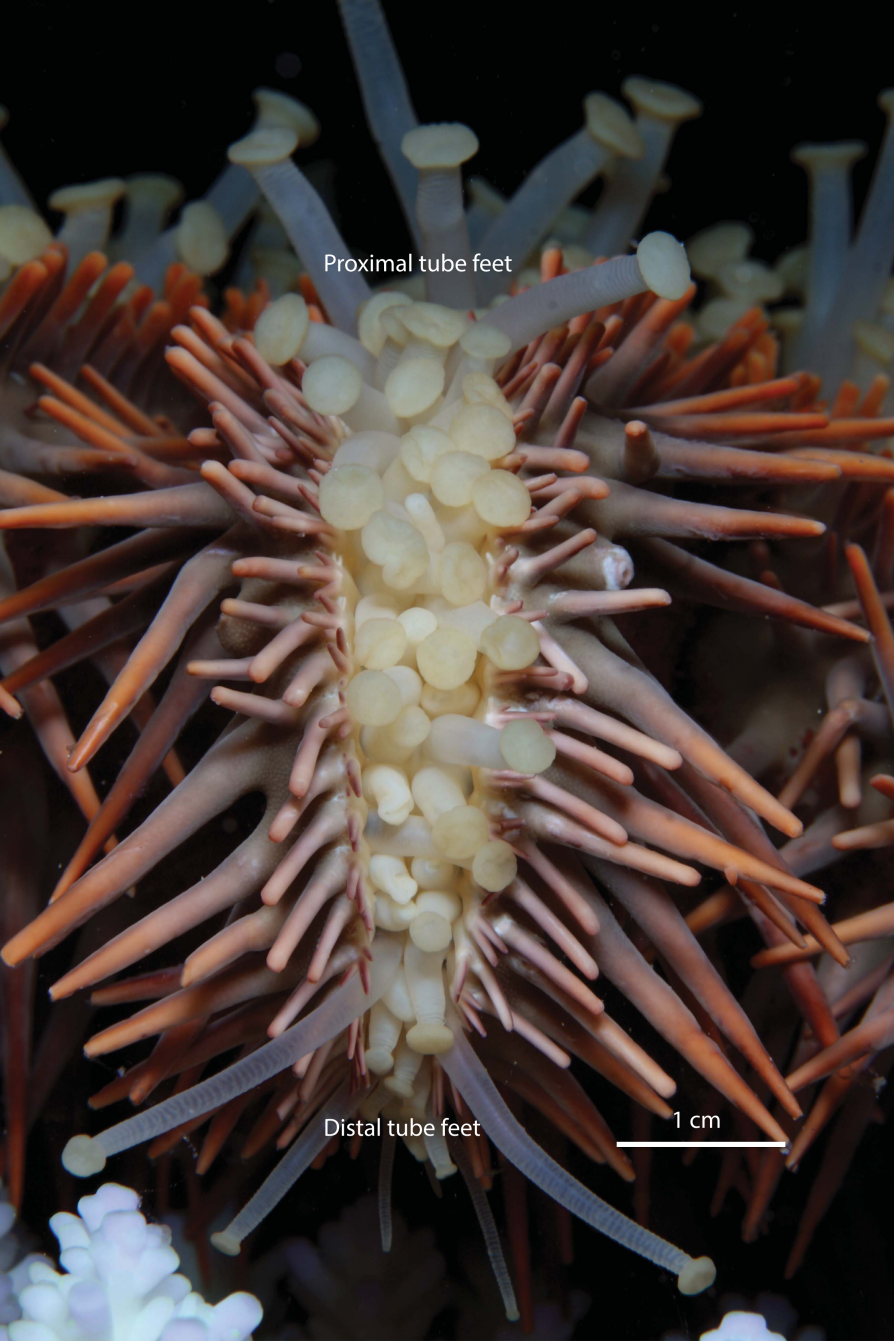
Oral (lower) surface of the arm of a crown-of-thorns starfish (*Acanthaster solaris*) showing variation in the size (length and diameter) of tube feet along the length of the arm. Photo by C.F. Caballes.

To test whether size-specific differences in movement rates of *A*. *solaris* were related to the size or number of tube feet, morphometric information was obtained for a subset of 42 individuals, ranging in size from 28–417 mm total diameter. Starfish were soaked in a solution of magnesium sulphate (10 g/ l MgSO_4_ in seawater) immediately prior to sampling to fully relax the individual tube feet. For each starfish, we sampled three randomly selected and non-adjacent arms, which were sectioned longitudinally to determine the number of tube feet. To account for missing tube feet, the number of pairs of tube feet was determined from counts of the number of dissepiments along the entire length of the radial canal, rather than directly counting tube feet. The length of tube feet was then measured at three positions along the length of each arm, recording the maximum extended length of tube feet at the base (within 1–3 cm of the oral opening), middle, and tip of the arm, giving a total of 9 measurements per starfish. The diameter of tube feet was not recorded, though there were noticeable differences in both length and diameter along the arm (Fig 1).

Morphological traits were quantified for freshly caught *A*. *solaris*, which had to be sacrificed, so we cannot directly relate movement rates to individual morphometrics. Rather, relationships between the number and size of tube feet versus the total diameter of crown-of-thorns starfish were established for a restricted subset of starfish (n = 42 starfish), all of which were originally collected from the same area on the northern GBR, and then used to estimate relevant trait values (based on their total diameter) for individuals used in movement trials.

### Movement experiments

To quantify mean and maximum rates of movement, individual *A*. *solaris* were placed into a large (5-m diameter) round tank. Three different substrate types were tested: i) a 5-cm thick layer of coarse (sediment-free) sand, which was collected from intertidal areas in inter-reefal back reef habitat; ii) a continuous layer of paving tiles which were coated in calcareous algae following extended deployment (up to 6 months) in reef environments before being air dried (reflective of consolidated carbonate pavement); and iii) coral rubble, mostly comprising an assortment of dead and air dried coral fragments (up to 10 x 10 x 5 cm) from experiments conducted at Lizard Island Research Station over the previous 12–24 months. To incite mostly unidirectional movement, a 1-m wide sheet of black plastic was attached to one section of the aquarium, which had otherwise white walls, following Beer et al. [29]. The black plastic sheet was intended to simulate the shade cast by a reef structure, representing a potential refuge detectable by *A*. *solaris* [18,29]. Movement trials were conducted during daylight hours and only when the sun was sufficiently elevated to directly light the bottom of the aquarium, from approximately 0830 to 1700. No live corals were placed within the experimental aquarium, such that movement recorded was motivated by inherent escape responses rather than prey acquisition.

Movement trials were initiated by placing a single *A*. *solaris* in the centre of the experimental aquarium. The movement of the starfish was then recorded using a GoPro camera mounted directly above the aquarium, which filmed continuously through successive movement trials. Individual starfish were left within the experimental tank until they either reached the outer perimeter of the tank, or stopped moving for >180-seconds. Starfish were randomly selected from the pool of captive individuals (n = 112), ensuring that each individual was tested only once on each substrate type. Some starfish were tested on multiple substrate types, but we did not explicitly account for individual identity and so cannot compare movement rates on different substrates for each individual. To test whether inherent escape responses were reflective of the maximum possible movement rates, we trialled the administration of an alarm cue (*sensu* [30]), made by emulsifying one arm of an otherwise uninjured and freshly collected starfish in 1-L of 0.2-μm filtered seawater. The freshly mixed alarm cue was then sprayed directly above the focal starfish within 2 minutes of placing the starfish in the experiment tank. Alarm cues were tested for approximately half of the individuals tested across each substrate. The entire experimental aquarium was emptied and flushed after use of alarm cues. Escape response from damaged conspecifics or tissue homogenates of conspecifics have been reported for echinoids [31,32], ophiuroids [33], and asteroids [34], but has never been explicitly tested for *Acanthaster* spp., where high rates of sub-lethal predation are presumed to occur based on high frequency of sublethal arm damage [35,36,37].

Videos were analysed using Adobe After Effects® CS6 (Adobe Systems Inc., San Jose, CA) to calculate mean and maximum rates of movement. Start time was defined as the time at which starfish began moving across the substrate and end time was determined when the individual either reached the edge of the substrate, stopped moving, or disappeared among the substrate (rubble only). To quantify movement rates, coordinates of the central point for each individual were recorded at 15-second intervals, and the distance between these two points was calculated and converted from pixels to cm, using measuring tapes laid across the bottom of the tank for calibration. Variation in locomotor capacity among individual starfish was analysed based on the mean rate of movement (cm/ second) averaged across all possible 15-second intervals for each starfish, and maximum rate of movement, which was the maximum distance moved across all 15-second intervals for each individual.

### Statistical analyses

Statistical analyses were conducted in the R Statistical Environment v 3.3.1 [38]. In order to assess the relationship between length and number of tube feet versus the mean diameter of individual, separate generalised linear models (GLM) were conducted (n = 44 starfish for each model; for each morphological parameter, three measurements were taken per starfish and the average was used in this analysis). To ensure that all assumptions were met, homogeneity of variance and normality were assessed through visual inspection of the residual and quantile-quantile (Q-Q) plots, respectively. Log-transformation of both length and number of tube feet were required to comply with these assumptions. The regression equations for length (y = 0.0639x – 0.8983) and number of tube feet (y = 0.0779x + 3224) were used to predict the length and number of tube feet in the starfish assessed for velocity analyses below.

Differences in the mean and maximum velocity (displacement in cm per 15 sec interval) were assessed using GLMs. Three morphological characteristics were assessed to determine the most appropriate parameter to explain variation in mean and maximum velocity, including mean starfish diameter, estimated length of tube feet and estimated number of tube feet. This was accomplished by selecting the most parsimonious model through the maximum likelihood estimation, by removing non-significant variables that did not result in a significantly larger Akaike information criterion (AIC). Estimated size and number of tube feet were calculated using the regression equations detailed above. The most appropriate morphological measure was included as a fixed factor in the velocity GLMs. Cue (alarm cue or control), substrate (paver, rubble, sand) and all relevant interaction terms were included as fixed effects (n = 216). Assumptions were checked through visual inspection of residual and Q-Q plots. Log transformation of both mean and maximum velocity were necessary to meet these assumptions. Significant differences between substrate types were further investigated using Tukey’s HSD post hoc tests, with non-significant interaction terms removed from the model.

## Results

**Fig 2:**
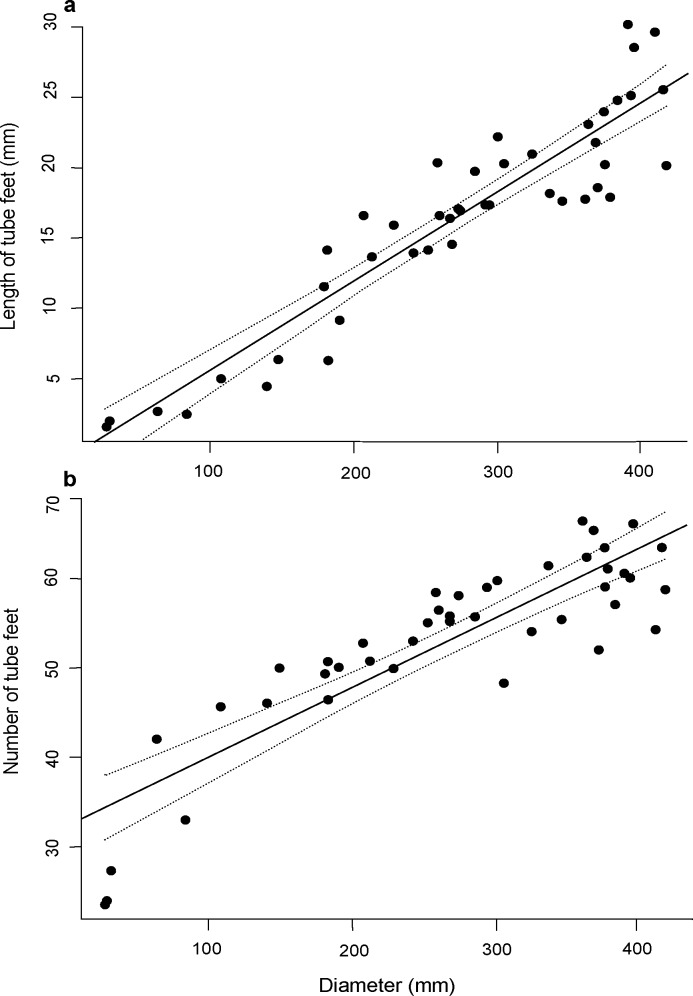
**Relationships for (a) average size of tube feet (mm), and (b) average number of tube feet per arm, with the total diameter of crown-of-thorns starfish (n = 42).** Solid lines indicate the line of best fit and dashed lines indicate 95% confidence intervals.

Tube feet of crown-of-thorns starfish varied noticeably in size (length and diameter) both within and among individuals. The tube feet were shortest near the mouth (14.43 ± 01.09SE mm) and increased in length toward the middle of the arms (17.44 ± 1.360SE mm). Tube feet at the distal portion of the arm were equivalent in length (16.07 ± 1.220SE mm) though noticeably thinner, to tube feet in the central portion of the arm (**Fig 1**). There were also marked differences in the length of tube feet among individuals ranging from a mean of 1.61 ± 0.20SE mm for a starfish that was 28 mm total diameter, up to 25.44 ± 0.55SE mm for a starfish that was 415 mm total diameter. The number of pairs of tube feet recorded across the 42 *A*. *solaris* ranged from a mean of 23.67 ± 0.33SE per arm for a starfish that was just 27 mm total diameter, up to 66.33 ± 0.33SE per arm for a starfish that was 360 mm total diameter. Both the length (GLM: F_1,41_ = 22743, p < 0.05) and number of tube feet (GLM: F_1,41_ = 10148, p < 0.05) were positively related to the total diameter of individual starfish (**Fig 2**). Moreover, the size and abundance of tube feet (independently or in combination) did not account for individual variation in the mean velocity of crown-of-thorns starfish (based on AIC comparisons of alternative linear models) any better than did total diameter. As a consequence, size-based differences in mean and maximum velocity were analysed using total starfish diameter.

**Fig 3:**
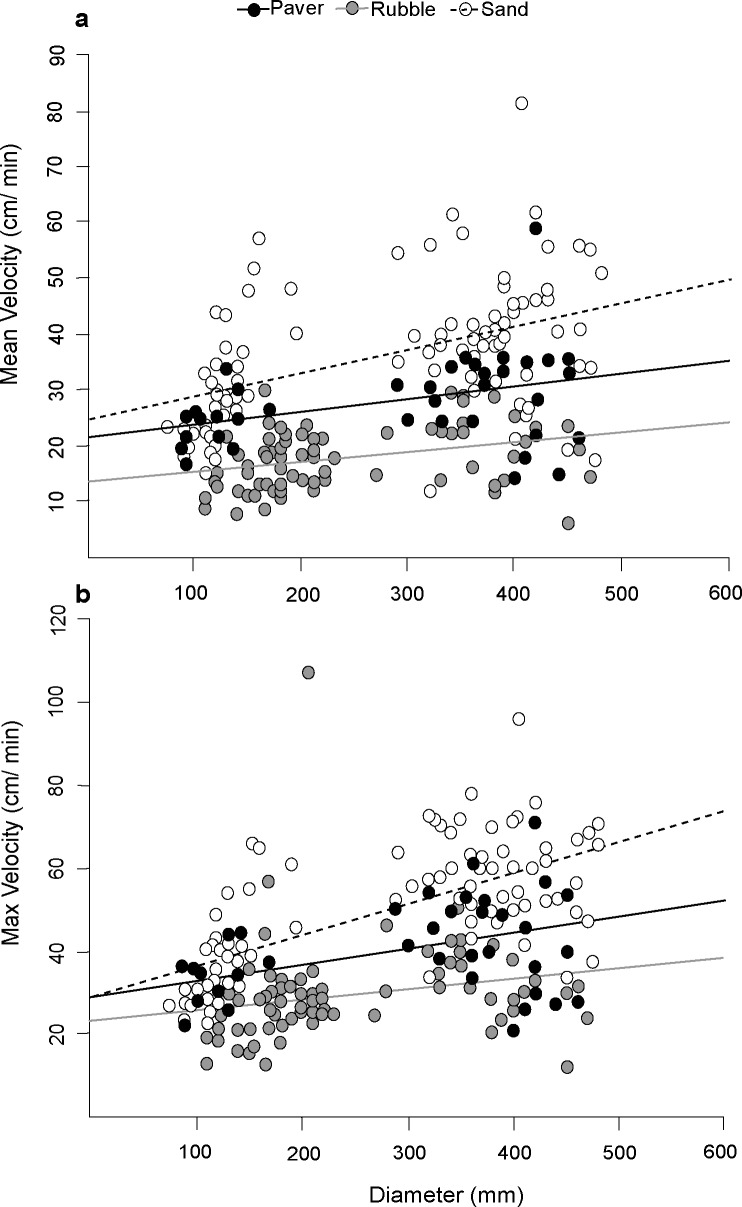
**Relationships for a) mean velocity and b) maximum velocity with the total diameter of crown-of-thorns starfish (n = 42).** Movement rates were quantified for three different substrate-types: paver (black circles), rubble (grey circles) and sand (white circles). Lines indicate the line of best fit for each different substrate type (paver: black line, rubble: grey line, sand: dashed line).

Most *A*. *solaris* started moving almost immediately (within 300 seconds) after being placed in the experimental tank, and once moving, most starfish (91.7%, n = 218) headed in an approximately linear path to the outer perimeter of the tank. Moreover, 75.0% of the crown-of-thorns starfish that reached the outer tank perimeter contacted the tank edge within the area covered by the black plastic sheet. Mean velocity varied significantly with the total diameter of starfish (GLM: F_1,214_ = 6178, p < 0.05; **Fig 3A**). The mean (±SE) rate of movement for smaller (<150 mm total diameter, n = 59) starfish was 24.0 ± 1.0 cm/ min compared to 33.4 ± 1.5 cm/ min for individuals >350 mm total diameter (n = 76). Substrate type also had a significant effect on mean velocity (GLM: F_1,212_ = 9848, p < 0.05; **Fig 3A**). Across all sizes, the mean velocity of *A*. *solaris* moving across sand (36.5 ± 1.3 cm/ min) was higher (Tukey’s test: p < 0.05) than on either rubble or consolidated pavement. Starfish had the slowest mean velocity on rubble (17.2 ± 0.6 cm/ min), with a significantly lower mean velocity than on sand or pavers (Tukey’s test: p < 0.05). There was no significant effect of alarm cues on mean velocity (GLM: F_1,211_ = 112, p = 0.29). None of the interaction terms (Diameter*Substrate, Diameter*Cue, Substrate*Cue or Diameter*Substrate*Cue) were significant (p > 0.05).

As with mean velocity, maximum velocity increased with mean starfish diameter (GLM: F_1,214_ = 9858, p < 0.05; **Fig 3B**). Substrate type also had a significant influence on maximum velocity (GLM: F_1,212_ = 6089, p < 0.05; **Fig 3B**). A significant interaction was detected between starfish mean diameter and substrate type (GLM: F_1,209_ = 341, p < 0.05; **Fig 2B**). The highest overall maximum velocity was recorded on sand. However, maximum velocity on sand was only significantly higher than rubble (Tukey’s test: p < 0.05). The difference in maximum velocity between sand and paver increased with increasing size of starfish, but was not significant (Tukey’s test: p > 0.05). A trend was indicated for higher maximum velocity on paver than rubble, but this effect was not significant (Tukey’s test: p > 0.05).

## Discussion

Crown-of-thorns starfish (*A*. *solaris*) placed in the centre of an open and well-lit tank almost invariably and immediately moved toward the outer perimeter of the tank, consistent with an inherent avoidance of open habitats [17,18]. The main exception to this were smaller starfish that were able to seek refuge beneath larger pieces of coral rubble, and did not, therefore, progress to the outer perimeter of the tank. Furthermore, most starfish headed directly towards the black plastic sheet (see also [29]), highlighting the importance of vision in orientation by crown-of-thorns starfish and their inherent preference for habitat structure [18,19]. Beer et al. [29] showed that *A solaris* exhibit functional differentiation of arms during movement, whereby one or more leading arms are bent upwards, while trailing arms are flattened, to maximise the visual field in the direction of movement. In addition to this conspicuous locomotor posture, we observed that some starfish (mainly those with damaged arms) tended to prioritise certain arms, consistently moving in the direction that the dominant and intact arm(s) were facing. Similarly, Rosenberg [39] also suggested that *Acanthaster* spp. may have a physiological anterior-posterior axis as demonstrated by the use of lead arms during non-random behavioural orientation. This tendency occurred even if it resulted in them circling around to head towards the black plastic sheet. Accordingly, *A*. *solaris* with either damaged arms or strong polarity in the dominance of certain arms may not have detected the black plastic refuge, potentially explaining why some starfish did not move towards the black plastic sheet.

Several different factors have been proposed to initiate movement by *Acanthaster* spp., including prey availability [11,40], presence of predators (see [29]), or adverse habitat and environmental conditions [21] that they can detect using olfaction [40,41], vision [18,19], or touch [42]. Maximum movement rates by starfishes are expected to occur in response to the presence or detection of potential predators (e.g. [22]). For *A*. *solaris*, olfactory cues of giant triton (*Charonia tritonis*) are reported to initiate rapid movement [43], though rates of movement during escape responses from this major predator have not been quantified. In the current study, exposure to the odour of a recently damaged *A solaris*, intended to represent a conspecific alarm cue, did not have any effect on the recorded rates of movement. This suggests that the rates of movement exhibited by *A*. *solaris* in our experimental arena were already maximised, owing to the fact that all starfishes were exhibiting a consistent escape response due to the prior handling. Alternatively, *Acanthaster* spp. may not respond to conspecific alarm cues, potentially due to saturation of such cues in high-density (outbreaking) populations. It should be noted that we did not attempt to establish any directionality of movement in relation to the putative conspecific alarm cues, but rather, specifically examined changes in the rate of movement of starfish. The importance and influence of conspecific alarm cues among asteroids may be context- and/ or species-specific, and, warrants further testing.

Mean and maximum rates of movement for crown-of-thorns starfish do increase with increasing body size. Previous measurements of movement rates, as it relates to foraging, have also shown that smaller *A*. *solaris* tend to be less mobile than larger starfish [26]. This is in contrast to the results of Mueller et al. [16], where movement rates did not vary significantly with body size. The reason for this divergence is unclear, but may be due to differences in the substrate type and size range of starfish used in experiments. Importantly, our study included a much greater range of sizes (75–480 mm total diameter) compared to Mueller et al. [16] (40–190 mm total diameter). The positive relationship between total diameter and length and number of tube feet shown here, suggests that size-related differences in movement rates may be driven, at least in part, by increases in the number or size of tube feet. Montgomery and Palmer [22] found that movement rates for the starfish, *Patiria miniata*, were largely determined by the arm length (relative to the size of the oral disk) and body mass. However, Montgomery and Palmer [22] showed that velocity actually decreased with increasing size of this starfish species, even though ambulacral groove area (≈ number or size of tube feet), scaled isometrically with body size. Other size-related changes in the morphology and physical capabilities of crown-of-thorns starfish may also influence movement rate. Several studies have suggested that it is the combined cross-sectional area of tube feet, relative to the body mass of individual starfish, which ultimately constrains the locomotor capacity of asteroids [14,22]. For crown-of-thorns starfish, apparent plasticity and individual differences in the number and size of arms caused by predatory injuries (e.g., [35,36]) may well contribute to overall differences in the number tube feet, and locomotor capacity, though this was not considered in this study.

Intuitively, substrate type must have a major influence on potential rates of movement by benthic invertebrates (e.g., [44]), but this study presents the first explicit test of effects of substrate-type on movement rates for crown-of-thorns starfish. We found that *A*. *solaris* moved slowest across rubble (**Fig 3**), which was the most structurally complex of the three substrate types tested. Three factors likely contribute to this. First, individuals must navigate a greater surface area to achieve the same degree of horizontal displacement with increasing topographic complexity of the substrate [27]. Second, tube feet adhesion strength is significantly higher when starfish are moving over rough surfaces [45]; therefore starfish may be slowed down on rough surfaces due to the need to break the stronger adhesive bond between their tube feet and the substrate, which could also explain why the effect of substrate was stronger in larger starfish (due to the greater number of tube feet and hence strength of attachment). Finally, the capacity of *A*. *solaris* to bend sufficiently to keep all tube feet in contact with the substrate will decrease with the substrate’s topographical complexity, and this effect should be greater for larger starfish. Interestingly, *A*. *solaris* exhibited consistently and significantly higher velocities on sand compared with flat, unstructured, consolidated pavement (**Fig 3**). Functionally, we’d expect movement rates to be somewhat constrained by the instability of sand and reduced adhesion of tube feet, limiting the capacity for starfish to pull themselves across the substratum. The greater velocities on sand versus pavement, might therefore, be due to motivation rather than physical capabilities, (see also [44]) or to the ease of breaking adhesion to sand surfaces.

In this study, *A*. *solaris* move fastest over sand. If average rates of movement measured here can be sustained, in combination with strong directionality, displacement distances of *A*. *solaris* could approach 520 m/ day for larger individuals. Even so, it would take weeks to months for *A*. *solaris* to move several kilometers between reefs. Also, the movement behavior captured in this study likely represents escape responses and maximum rates of movement that can only be sustained for short periods. Large-scale connectivity, and sequential initiation of outbreaks among widely separated reefs, is unequivocally achieved mostly through larval dispersal [3,46, 47,48] and there is limited evidence that adult *Acanthaster* spp. actually move between reefs. Pearson and Endean [15] have observed *A*. *solaris* crossing large expanses of sand between patch reefs, but not *en masse*. However, Suzuki et al. [49] reported mass strandings of crown-of-thorns starfish in shallow inter-reef sand flats around Ishigaki Island, southern Japan. Following depletion of corals, starfish aggregations were observed moving across shallow sand flats, presumably in search of food, but were exposed and stranded at low tide [49]. This may be a special case for reefs within lagoons around islands. For the most part, inter-reef movements probably occur only very rarely and contribute little to the overall spread of outbreaks, but will be disproportionally important in terms of structuring management and control actions that can reduce the impact of starfish at local scales [50].

Information on potential and realised movement by *Acanthaster* spp. is important for optimising spatial and temporal aspects of localised culling and removal activities (e.g., [28]), at the sub-reef, reef and reef-complex scales. On the GBR, for instance, extensive time and effort is invested in preventing coral loss at select locations (mostly high-value tourism locations) through recurrent culling of *A*. *solaris* [50], by injecting individual starfish with a bile salts solution [51]. To be effective and efficient, ecological control programs must be structured at the spatial and temporal scales relevant to the scales at which individual organisms move through the landscape [12]. Most notably, the rate at which individuals can move from uncontrolled to controlled portions of a reef and the scale of those movements will directly affect the efficacy with which the control program can protect sites of economic importance, and has important ramifications for the size of buffers required around areas to be protected, and the frequency of revisitations to each site [52]. This study goes someway to establishing potential movement rates of starfish of different sizes across different substrates, but this information will need to be supported with field measurements of movement rates.

In conclusion, this study has demonstrated that both body size and substrate-type modulate potential movement rates of *A*. *solaris*. If average rates of movement measured here can be sustained, in combination with strong directionality, displacement distances of crown-of-thorns starfish could range from 150–520 m day^1^, depending on the size of the starfish and prevailing substrate. While these movement rates are estimated from short-term laboratory studies (to control for many potentially confounding factors), this work enables a much better understanding of the potential capacity for movement of *A*. *solaris* within and among coral reef habitats. Actual displacement and redistribution of *Acanthaster* spp. within reef habitats will likely occur much more slowly, largely owing to limited periods of sustained and unidirectional movement. Moreover, starfish moving through reef environments are likely to opportunistically feed on corals in their path, further reducing rates of displacement. Parallel studies to quantify movement rates under field conditions (at time scales of days to months) are warranted, and are underway.
